# Pathway Analysis: State of the Art

**DOI:** 10.3389/fphys.2015.00383

**Published:** 2015-12-17

**Authors:** Miguel A. García-Campos, Jesús Espinal-Enríquez, Enrique Hernández-Lemus

**Affiliations:** ^1^Computational Genomics, National Institute of Genomic MedicineMéxico City, México; ^2^Complejidad en Biología de Sistemas, Centro de Ciencias de la Complejidad, Universidad Nacional Autónoma de MéxicoCiudad de México, México

**Keywords:** pathway analysis, systems biology, high-throughput biological data, pathway-topology, functional class scoring, over representation, bioinformatics

## Abstract

Pathway analysis is a set of widely used tools for research in life sciences intended to give meaning to high-throughput biological data. The methodology of these tools settles in the gathering and usage of knowledge that comprise biomolecular functioning, coupled with statistical testing and other algorithms. Despite their wide employment, pathway analysis foundations and overall background may not be fully understood, leading to misinterpretation of analysis results. This review attempts to comprise the fundamental knowledge to take into consideration when using pathway analysis as a hypothesis generation tool. We discuss the key elements that are part of these methodologies, their capabilities and current deficiencies. We also present an overview of current and all-time popular methods, highlighting different classes across them. In doing so, we show the exploding diversity of methods that pathway analysis encompasses, point out commonly overlooked caveats, and direct attention to a potential new class of methods that attempt to zoom the analysis scope to the sample scale.

## 1. Introduction

Pathway Analysis (PA), also known as functional enrichment analysis, is fast becoming one of the foremost tools of Omics research. The main purpose of PA tools is to analyze data obtained from high-throughput technologies, detecting relevant groups of related genes that are altered in case samples in comparison to a control. In this manner, PA methods seek to overcome the problem of interpreting overwhelmingly large lists of important, but isolated genes detached of biological context, which are the main output of most basic high-throughput data analysis, as differential expression analysis. PA methods provide meaning to experimental high-throughput biological data (HTBD) thus facilitating interpretation and subsequent hypothesis generation. This has been achieved on the basis of coupling existing biological knowledge from databases with statistical testing, mathematical analyses and computational algorithms.

PA methods possess a broad range of applications in physiological and biomedical research. These methods aim is to help the researcher discover what biological themes, and which biomolecules, are crucial to understand the phenomena under study, given the HTBD analyzed. In turn, the clues that provides a PA enables the researcher to generate new hypothesis, design subsequent experiments, and further validate their findings. PA methods have helped researchers in the identification of the biological roles of candidate genes, selected to design new therapies for cancer, circumventing collateral damage to healthy cells (Folger et al., 2011). Another instance is the determination of similarity and dissimilarity, at a molecular level, between sample groups, as in the comparison between cell lines and tumor samples (Heiser et al., 2012). Such kind of analyses may help researchers understand heterogeneity phenomena in different research contexts. Yet another example is the use of PA methods to examine the biological function of gene modules, not yet validated sets of genes thought to be related between them, as in the analysis of genes that fluctuate in response to natural variations, like seasons (Dopico et al., 2015). Although all these applications have succeeded in specific goals, the use of PA methods may be as wide and complex as the creativity of their users.

However, despite the recent spotlight and wide usage PA has gained in recent years, overlooking of the key elements that compose these methods is also common. Often users neglect details concerning the proper application of the methods, their caveats, and the existence of different PA methods. In this regard it is essential to review the foundations and diversity of the PA methods, and acknowledge their capabilities and caveats.

There are several elements needed to perform a PA. First of all, quantitative data representative of the cell biology is needed. This information is generated through the use of Omic technologies as: RNA-microarrays, tandem mass spectrometry, and RNA sequencing. Secondly, an approach able to analyze such substantial amount of data is mandatory. Systems biology is an emerging field of research that enables the study of living organisms as systems, opposing reductionist approaches (Hartwell et al., 1999; Kitano, 2002). Systems Biology uses Omic data as the main input of its analyses. In third place, molecular biological knowledge stored in data bases is required for the analysis to be performed, guiding PA methods to search relationships between the generated Omic data and known biological themes. Finally, the computational machinery needed to accomplish PA is needed. It consists mainly in statistical testing of the biological themes vs. the data, and other mathematical algorithms that seek to extract relationships between the data and previous knowledge.

The purpose of this review is to act as an introduction to the aforementioned foundations, and other guidelines for understanding PA. We will give an overview of different kinds of methods, their capabilities and limitations, and acknowledge the emergence of a new kind of methods. At the same time, we will provide an up-to-date panorama of available methods and resources for PA users to pick from. This should allow for a fast and clear initiation in the use of these promising research tools.

## 2. Foundations of pathway analysis

### 2.1. Omics. making molecular life measurable

With the first bacterial genome sequenced in 1995 (Fleischmann et al., 1995), followed by the competition and optimism generated by the Human Genome Project, reporting its first drafts in 2001 (Lander et al., 2001; Venter et al., 2001), the spark that ignited the beginning of an era of massive biological information gathering was lit in the dawn of this century.

Nowadays genomes have become our roadmaps, providing a guide to subsequent discoveries in human (and other species) biology. At present, more than 35,500 prokaryotic and eukaryotic genomes are at public disposition in the National Center for Biotechnology Information site, as completely sequenced or in progress builds. Since the inception of the term “genomics” in academic literature, research in this field has changed and evolved over dramatically different aspects of human knowledge (Siqueiros-García et al., 2014). These facts evidence how modern life sciences research will be dependent of this ever growing biological information gathering, which expands in a multidimensional way, and at an increasingly detailed manner.

Molecular analysis of living organisms has pushed technological advancements to develop techniques that measure the status of key biomolecules, able to inform how living organisms function in a molecular manner, namely: DNA, RNA, proteins and small molecules of diverse nature. Efforts into analyzing such components in aggregate, led to the development of the research fields that altogether are widely known as Omics (Weinstein, 2002; Ge et al., 2003; Westerhoff and Palsson, 2004). Current Omic research includes not only the analysis of information from DNA sequences, but also characterizes different sets of biomolecules, for instance: global profiles of RNA sequences (transcriptome), of proteins (proteome), of DNA methylation events in a genome (methylome), of metabolites (metabolomics) among others. Data generated by all these Omic approaches is generally regarded as HTBD.

Our current theoretical framework in Omic research has developed around the assumption that analysis of HTBD information should help to understand the underlying mechanisms that determine biological phenotypes. This assumption has proven effective in many instances, but interpretation of HTBD is not straightforward, as we unveil how truly complex life is (Amaral and Ottino, 2004).

Computational testing of Omics data, by a naïve approach, would take more time than the age of the universe itself (Huang, 2000). For instance, taking 25,000 as the number of genes in the human genome, and modeling its states as only “on” or “off” would lead to the astronomical number of 5.6 × 10^7525^ possible gene profiles. That is why, a highly efficient mathematical approach that couples biological knowledge with HTBD is needed, reducing dramatically the space of possible hypotheses to be tested. In this regard, PA has established to provide one possible answer to this new century problem.

### 2.2. Systems biology. an integrative approach in molecular biology

Human engineered machinery, which components can be as numerous as the 3 × 10^6^ parts of a Boeing 747-400, can be truly complicated, but each of their components' role is well-defined and governed by understood rules. In contrast, living organisms “machinery” has proven to be even more complex to understand, having large number of components that may act by following dynamic rules that are not well-understood. Thus, the study of isolated biological components is not enough to understand biological systems, leaving many questions still open, for instance, the ones related with the functioning of the human brain and the development of multifactorial diseases (Amaral and Ottino, 2004).

The study of life at a systems level requires information capable of reflecting the nature of whole biological phenomena, and an approach able to incorporate such information. As already mentioned, biomolecular information that intends to represent organisms as systems is obtained through Omic approaches in the form of HTBD. Analysis of such data, with an integrative approach, may enable the elucidation of not yet reported properties and underlying mechanisms. Systems biology is a research area whose central task is to integrate many levels of biological information, from DNA to ecosystems, into predictive mathematical models that in turn can create coherent hypothesis to be experimentally tested generating new knowledge. This coupling of approaches can generate a cycle between discovery-driven and hypothesis-driven science (Ideker et al., 2001). In this manner HTBD and systems biology can act as the marble and chisel of an elaborated process of knowledge generation.

Difficulties arise from the integration of myriads of information variables and sources into coherent models that enable to capture biological meaning. Two of the main problems to tackle in HTBD analysis are: the curse of dimensionality, caused by a greater number of variables than of samples; and the development of algorithms that effectively integrate and analyze biological information, embracing current and upcoming knowledge. PA has developed and established as a plausible answer to cope with both of these issues.

### 2.3. Pathways as functional biological units

Although the pathway concept has recently boomed with attention, the idea of a set of genes functioning to accomplish a specific task, has been around since the first genetic maps were constructed in the 1950's (Lawrence, 1999), observing non-random associations of genes contributing to single functions or phenotypes, as in *Neurospora* biosynthetic pathways (Barratt et al., 1954) or as in the early developmental genes from *Drosophila* (Lewis, 1952).

As one can note, rarely in nature a single molecule comprise an entire functional trait, as well as a single function (Hartwell et al., 1999). To understand the complexity of biological organisms at a molecular level, many simplifications have been drawn. The first of this is the acknowledgment of change in phenotype at the single-gene-level. This is, that a given modification on a single gene, would lead to a specific change in an organism, e.g., mice lacking Apo B gene have infertility problems for heterozygotes and embryonic lethality in homozygotes (Huang et al., 1995). Although the above approach has been fruitful, and constitutes an important part of our biological foundations, it is not ideal for a bulk analysis of HTBD.

A helpful proposal in the trouble of analyzing HTBD, given by Hartwell et al. (1999), is the recognition of functional “modules” as a critical level of biological organization. A module is a discrete entity whose function arises from the interactions among its components and it is separable from that of other modules (Hartwell et al., 1999). In line with this proposal, a convenient addition is to conceptualize these modules as networks. A network is defined by a set of items, called nodes, with connections between them, called edges (Newman, 2003). Generally nodes in biological networks would represent biological physical entities, such as proteins, nucleotides, carbohydrates, and small metabolites among others, while edges would represent a relationship between biological entities, for example, binding, activation or inhibition.

Though separable as units of study, Hartwell's modules, here on referred as *pathways*, are in reality connected and intertwined into a single large network that conforms all the possible interactions among life components. The nodes of pathways, here on termed as *pathway components*, may be linked to different pathways at the same time, and its individual function may vary according to its cellular context, for instance, human genes as ras, myc, rho, and NF-κB either stimulate survival and cell growth, or induce apoptosis (Huang, 2000). In this manner, specification of biological interactions increments a level of intricacy, both in the analysis and elucidation of function.

Networks have established as a useful representation for pathways. Networks and pathways have been for a long time portrayed in textbook descriptions of regulatory, metabolic and signaling processes. Although visually appealing, pathways have not only helped people understand the theoretical complexity of molecular models, at the same time (aided by the interdisciplinary framework of systems biology) they have constituted a scaffold for the development of new tools of study for complex biological phenomena.

### 2.4. Pathways as informatics units

The acceptance of the pathway concept has led to many publications that describe them; but literature knowledge is hard to integrate one paper at a time. In this sense, the pathway concept has been transported from a literature scheme to an informatics framework, in the form of pathway data files compiling the information from different sources into a single *model*.

A pathway can be represented then as a data file, specifying which biological components are related in a common biological theme, and in some cases, the relationships they keep. Pathway data can be broadly divided into two different kinds: Gene-sets and Pathway Topology. Gene sets are simple lists of biological components, in which the listed entities share a common biological theme. In contrast, pathway topology not only lists the components of a pathway but also describes the interactions between them, showing *who* is interacting with *whom*, and how do these interactions happen, hence turning into a more detailed description of how do the molecular components of a biological process are working together.

**Figure 1:**
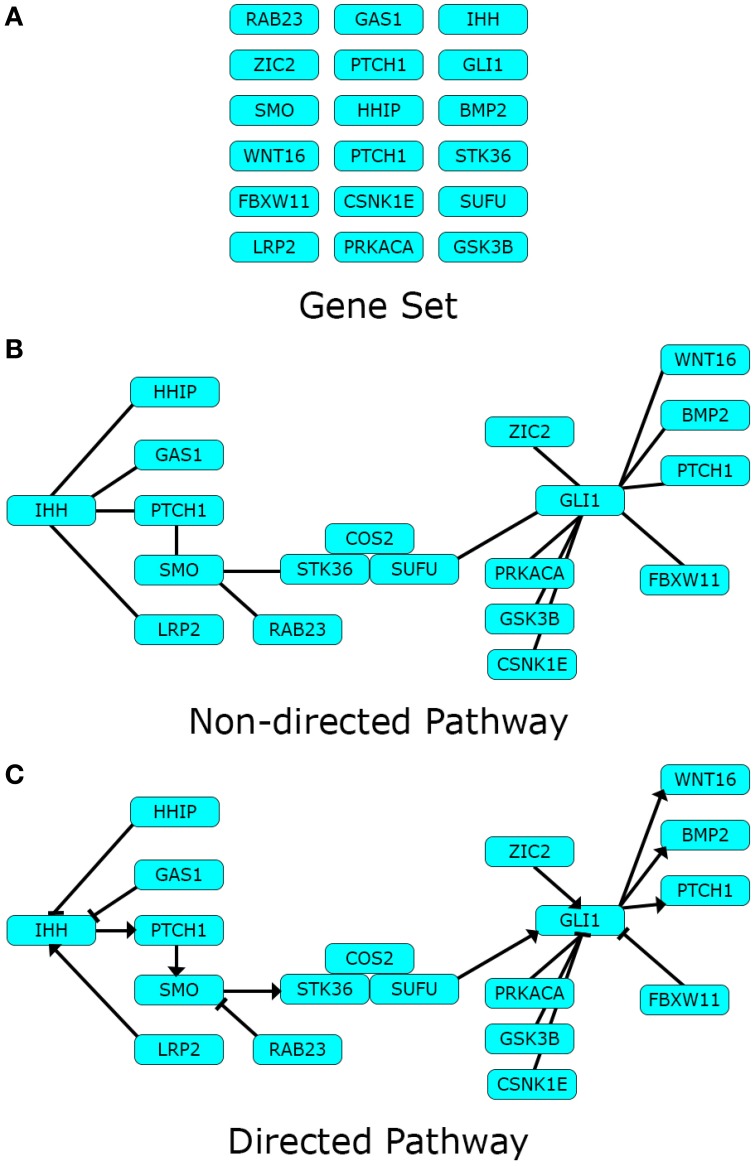
**Pathway data-types**. Different types of pathway data can represent one pathway in different levels of detail. Here we use the Hedgehog signaling pathway modified from Kyoto Encyclopedia of Genes and Genomes (KEGG) as toy example. **(A)** Gene sets are lists of biological components pertaining a definite biological theme; while **(B)** Non-directed pathways describe the existence of definite interactions between the same components in the form of a network; finally **(C)** directed pathways disclose the character of the interactions in the network. Arrows depict an activating impact from the pointer component over the pointed one, and blunt edges an inhibiting one.

Pathway topology may contain additional information relative to the connections between components. The character of the interactions between components can be directed, if the presence of a certain component is known to affect another in a given sense. Additionally, the character of a directed interaction can be positive or negative, depending on whether the role of the pointer component is to activate or inhibit the pointed component. An example of these three pathway representations: Gene set, non-directed pathway, and directed pathway can be found in Figure 1.

Although some authors have declared that gene sets are not legitimate pathways (Mitrea et al., 2013), gene sets have proven useful for analysis purposes, and the understanding of PA is not complete without taking them into consideration. In this sense, this review subsequently accounts all three models as pathways.

### 2.5. Pathway databases

Efforts in structuring biological knowledge on pathways have resulted in the generation of Pathway Databases (PDBs), also referred as “knowledge bases,” which condensate current biological knowledge of molecular interactions in pathway data collections. PDBs usually retrieve and structure data from different sources. Generally, experimental evidence is curated from literature, and computational analyses are carried out by the project itself to infer the possible functions of homologous biomolecules. Additionally cross-reference of data between similar databases is generally performed. For example, Reactome (Vastrik et al., 2007) database annotations are manually curated from literature by expert biologists in collaboration with their editorial staff, and cross-referenced it with several other resources, as primary literature, and other pathway related databases (Croft et al., 2010). Currently hundreds of PDBs projects are established, and actively annotating biological knowledge, each one in specialized contexts.

Pathway-database current catalog is truly abundant and diverse, ranging in species focus, curation approach, kind of pathways and interactions covered, among other differences. A mandatory first stop for looking into the prospect of Pathway Databases is the pathguide.org website (Bader et al., 2006), currently listing 547 pathway-related databases, divided into 9 categories according to the kind of interactions they focus on, accounting for more than 2.5 million pathways in total. A list of commonly used PDBs and the focus category they fall can be found in Table 1.

**Table 1:** **Pathway databases**.

**PDB Name**	**Pathway focus**	**URL**	**Y.O.R**.	**Standard formats**
EcoCyc	M,S	biocyc.org	1995	SBML, BioPAX
KEGG	M,S,D	kegg.jp	1996	BioPAX
RegulonDB	GR	regulondb.ccg.unam.mx	1997	BioPAX
MetaCyc	M	metacyc.org	1999	SBML, BioPAX
STRINGDB	PPI	string-db.org	2000	PSI-MI
PANTHER	S,D,PS	pantherdb.org	2004	SBML, SBGN, BioPAX
Gene Ontology	PPI,M,S	geneontology.org	2000	
REACTOME	M,S,D	reactome.org	2005	SBML, SBGN, BioPAX, PSI-MI
MSigDb	M,S,GR	broadinstitute.org/gsea/msigdb	2005	
Ingenuity Knowledge Base^*^	PPI,PCI,M,S,GR,D	ingenuity.com	2005	
NCI PID	S,D	pid.nci.nih.gov	2006	BioPAX
WikiPathways	M,S,D	wikipathways.org	2008	BioPAX
Small Molecule Pathway DB	M,S	smpdb.ca	2009	SBML, BioPAX
ConsensusPathDB	PPI,PCI,M,S,GR	consensuspathdb.org	2009	BioPAX, PSI-MI
Pathway Commons	PPI,PCI,M,S	pathwaycommons.org	2010	BioPAX

*A brief example of the diversity of available PDBs found online. The second column shows the kind of biological focus pursued by each database: (PPI, protein-protein intereactions; PCI, protein-compound interactions; M, metabolic; S, signaling; GR, gene regulation; D, diagrams; PS, protein sequence). The last column addresses the standard pathway languages adopted to provide data. Additionally, in the third column the links to web sites are supplied. YOR, Year of release*.

* *Commercial database*.

Since their development, PDBs have allowed a different approach for biological knowledge gathering, use and discovery. Frequently, different PDB projects work in conjunction between them, sharing their information, generating fluxes of information, cross validating their data, and converging in coherent manners. This has permitted an increasingly easier and automated data retrieval process, speeding up the knowledge-discovery process.

However, an important feature to check when using information from different PDBs, is the pathway ontology they have adopted. Pathway ontologies are the notion or definition of “pathway” used by each PDB. Different pathway ontologies are best suited for different tasks, and the use of different pathway concepts can lead to different outcomes in computational studies (Green and Karp, 2006). However, a way to manage the information from different PDBs, is using a unified ontology.

Unifying ontologies across PDBs is accomplished through the use of pathway standard languages. These are standard formats that seek to facilitate the exchange of pathway data between PDBs and PA tools. A gold standard for pathway annotation in PDBs does not exist, but most pathway data is based in the Extensible Markup Language (.xml) or in plain text (.txt) formats. Encoding pathways in such formats makes them readable for both humans and machines. Examples of these standard languages are: the Systems Biology Markup Language (SBML; Hucka et al., 2003), the Systems Biology Graphical Notation (SBGN; Le Novere et al., 2009), or the Biological Pathway Exchange (BioPAX; Demir et al., 2010). An overview of the standard languages some PDBs have adopted can be found in Table 1. Each one of these formats has different characteristics and uses, although efforts are made to ensure translatability across languages. Literature regarding their use and comparisons between them can be found in Strömbäck and Lambrix (2005), Suderman and Hallett (2007), Wierling et al. (2007), Bauer-Mehren et al. (2009).

Additions and corrections to PDBs are made periodically, thus increasing the quality and coverage of their biological knowledge. Some databases are able to update their information in a frequent basis, to maintain pace with new discoveries. For example the KEGG database (Kanehisa, 1997) updates its data in a weekly basis, but other PDBs do it less often, as Gene Ontology (Ashburner et al., 2000), which updates its data in a monthly basis. Nevertheless, some PDBs fail to update their information in a regular basis, thus they become obsolete overtime, yet are employed by users of PA tools, caution is suggested when using outdated PDB data.

As information of PDBs seek to comprise our current biomolecular knowledge, it is difficult to ascertain their quality. Every annotation that PDBs contain is supported by different kinds of evidence of differing regarded quality. Manually curated experimental evidence is regarded of the highest quality, whilst computationally inferred evidence and electronically annotated evidence are commonly regarded of lower and lowest quality respectively. A common practice in control-quality of pathway information, when manually extracting data from PDBs, is to discard annotations from electronically inferred evidence. Although the assumption that electronically inferred annotation is of lower quality has not been robustly proven (Rhee et al., 2008), and doing so reduces dramatically the coverage of PDBs.

Different authors have showed that no PDB is comprehensive, and suggest data integration from different PDBs for improved quality of pathway data (Khatri and Drăghici, 2005; Adriaens et al., 2008; Bauer-Mehren et al., 2009). Nevertheless, integration procedures may prove to be extremely challenging, as an analysis of some PDBs has shown low levels of consistency and compatibility among them.

Coverage of PDBs is another important consideration to have in mind when performing PA, and considering PDB data quality. This is, the proportion of aggregated biological components described in all the pathways from a PDB with respect to a reference list of components. For example, one of the most comprehensive public composite PDB, Pathway Commons (Cerami et al., 2011), with the aggregated information from 22 PDBs, currently has a coverage of 17,439 gene symbols from the 39,241 accepted ones, this is roughly 45% of the total symbols from the official HUGO Genome Nomenclature Committee (HGNC) registers. Using the most up-to-date and complete information is important for optimal information extraction from experimental data. Accomplishing this is not only a call for users to use the best PDBs available, but for potential contributors to improve the coverage and knowledge invested in databases. Most public databases encourage open collaborative efforts, with corresponding revision of the shared data. All such improvements in PDBs will ultimately lead to a faster knowledge discovery and application of the biological knowledge.

## 3. Pathway analysis methods

Because of the large amount of biological knowledge stored in PDBs, it may be as difficult to grasp for the human mind as happens with the information from HTBD. A bridge between technology of Omics platforms and knowledge sources is created with the help of PA methods. Though PA methodologies are diverse in statistical approaches, and computations performed, some authors have proposed general workflows they all work along (Ackermann and Strimmer, 2009; Mitrea et al., 2013). These workflows can be summarized in three phases: Input, Analysis, and Output.

### 3.1. General *pathway analysis* workflow description

#### 3.1.1. Input phase

This phase consists in the arrangement of all things necessary to start the analysis, and its input in the PA method. Besides the important preliminary choice of a PA method, this phase consists in the preparation of two datasets. The HTBD to be analyzed, and the pathway data, extracted from PDBs, in which the analysis will be performed. These two data sets will be the input to virtually any method we choose. A helping analogy can be the following: a haystack from where we want to find needles. Organizing the haystack and recognizing characteristics of the needles will make easier our task.

A key step in the setup of the analysis is the selection of null hypothesis. These can be broadly classified in three kinds (Ackermann and Strimmer, 2009) and briefly explained as follows: “competitive null hypothesis” (Q1): the genes in a pathway are differentially expressed as often as the rest of the genes; “self-contained null hypothesis” (Q2): no genes in a pathway are differentially expressed; and the “nested null hypothesis” (Q3): the false discovery rate (FDR) estimates for genes in a pathway are the same estimates for all genes (Efron and Tibshirani, 2007; Ackermann and Strimmer, 2009; Heinig, 2011). Using a null hypothesis over others should depend on the biological interpretation given to it (Goeman and Bühlmann, 2007), and ultimately accounting this will be crucial for the PA method choice. Discussion about the null hypothesis employed in PA, their importance, and validity can be found in Allison et al. (2006), Goeman and Bühlmann (2007), Nam and Kim (2008), Glazko and Emmert-Streib (2009).

Experimental dataset preparation is already considered into most common preprocessing stages of HTBD preparation, however, one may need to perform particular preprocessing procedures before introducing HTBD into the workflow of most PA methods. Common preprocessing steps may include: normalization of data across samples; batch effect correction, if the data's origin is from different but comparable experiments; collapse data to unique gene identifiers, most commonly gene symbols; gene selection by differential expression analysis; experimental vs. control data labeling; etc. A broader description of these preprocessing procedures can be found in Hung et al. (2011).

Pathway information dataset preparation must encompass: the HTBD to be analyzed and the biological hypothesis we want to obtain. Searching for PDBs that cover information about the specific biological components we are working with is also mandatory. Species-specific PDBs are examples of high relevance, as most general use databases will have pathways pertaining to *Homo sapiens* or a consensus pathway between different species. For example, to analyze the pathways of *Saccharomyces cerevisiae*, a suitable PDB would be the Saccharomyces Genome Database (SGC; Cherry et al., 1998).

#### 3.1.2. Analysis phase

The Analysis phase consists of all statistical and mathematical computations, performed by the PA method, with the aforementioned datasets. Although the algorithms used are diverse, they share commonalities and are guided by the same approach. Fundamentally all PA methods statistically test the possibility that any given pathway is represented by the HTBD, resulting in the identification of pathways that have the smallest possibility of being represented by randomly generated data.

PA software can be generally found in three different manners: as stand-alone software, as web-based applications and as programming packages. The first two classes are frequently easier to use, as they do not require extraordinary analytical skills or programming abilities. The last kind, is mostly coded in the R and Python languages, and shared openly through the BioConductor (Gentleman et al., 2004) and GitHub (Dabbish et al., 2012) projects. The main benefit of using PA software developed as programming packages, is the customization potential of every part of the analysis, as well as the possibility of automation via scripted analysis pipelines. Choosing between software platforms may be a mixed question between user skills, and cost-benefit ratio of time invested in arranging all things necessary to run the analysis.

In opposition to black box approaches, PA methods are commonly well-explained and documented in their publications, an important fact since understanding what each method calculates is essential to generate relevant hypothesis. This ultimately should reduce computational costs, as well as experimental verification costs and human time.

#### 3.1.3. Output phase

**Figure 2:**
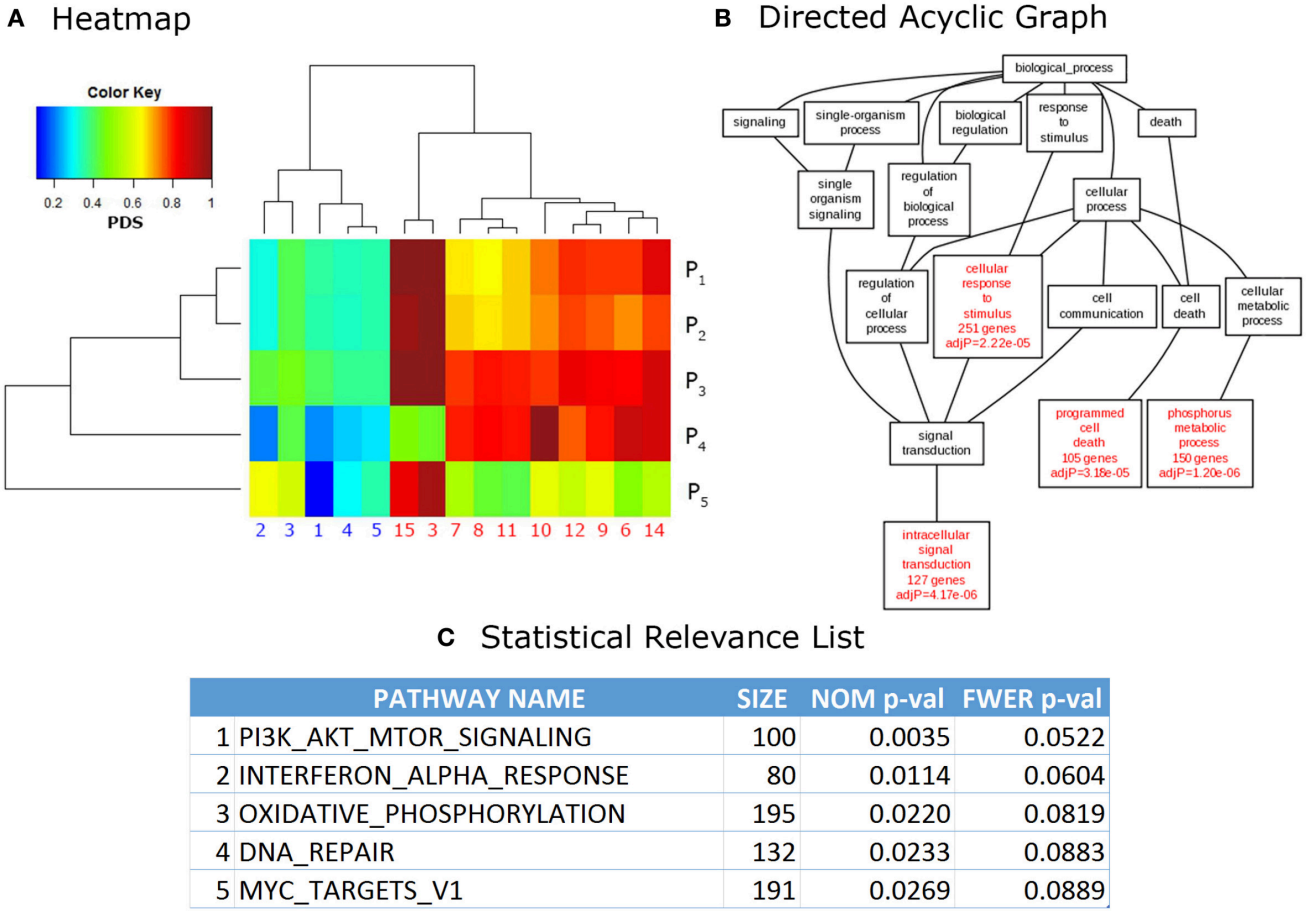
**Common outputs of PA tools**. **(A)** Heatmap. Each cell of a heatmap represents a numerical value with a color code. In this case lower values are represented in blue, while higher values turn to red. This example shows data analyzed with Pathifier (Drier et al., 2013), the phenotypic information for data used in these calculations is irrelevant, it was used only for illustrative purposes. **(B)** Directed Acyclic Graph (DAG). DAGs can be used to represent partially ordered items. In this case, relevant GO categories are highlighted in red with their respective confidence values. This DAG is a partial result from the example data provided in WebGestalt (Zhang et al., 2005) website. **(C)** Statistical Relevance List. This kind of lists is the most common output in PA methods. In it, the statistical significance of the top pathways ranked on their *p*-values (NOM = nominal, FWER = Family wise error rate corrected, Size = size of the pathway) is shown. This is an example of data analyzed through Gene Set Enrichment Analysis (GSEA; Subramanian et al., 2005), used only for illustrative purposes.

Finally, the output phase consists in visualization and analysis of the results. More important, they are presented in a ranked list of relevant pathways, where the top pathways are often ordered by confidence values such as *p*-value or the multiple testing corrected *q*-value. Other formats (used mainly for visualization of results) are in the form of directed acyclic graphs in which relevant categories are hierarchically ordered according to its relationship, for example, within the Gene Ontology categories. Heatmap formats are also popular for visual interpretation of results as pattern generation across related pathways and samples is easier to observe in this way. Additionally, most web-based and stand-alone software provide links to web pages in PDBs, as well as other online resources, for easier integration of the results. Figure 2 shows examples of outputs for different PA tools.

### 3.2. Classification of *pathway analysis* methods

Though diverse in computations and data used to perform the analysis, PA methods share commonalities between different methods. Hence many authors have proposed different systems to classify them (Nam and Kim, 2008; Ackermann and Strimmer, 2009; Glazko and Emmert-Streib, 2009; Huang et al., 2009; Khatri et al., 2012). Evolution and diversity of PA methods can make any classification questionable, but every attempt has helped to grasp the potential use of these methods as a whole.

A key methodological difference that divides all PA methods is the use of univariate or multivariate statistics, performed in the analysis phase. Univariate analyses account for one variable at a time, in this case genes or other biomolecules, while multivariate analyses consider more than one variable simultaneously. Examples of PA methods that use an univariate approach can be found in Zeeberg et al. (2003), Boorsma et al. (2005), Subramanian et al. (2005), and methods that use an multivariate approach can be found in Goeman et al. (2004), Kong et al. (2006), Hummel et al. (2008), Jacob et al. (2012).

Intuitively it is expected that multivariate analyses, testing the joint distribution of genes, accounting for interdependencies among them, is of greater statistical power than univariate analyses, testing differences only between the marginal distributions (Glazko and Emmert-Streib, 2009). However, evaluation performed on simulated and real biological data showed that both approaches have similar statistical power at the less severe significance cutoffs (*p* ≤ 0.05), and notably, univariate approaches are more powerful at more severe significance cutoffs (*p* ≤ 0.001). Nevertheless, every method found different sets of pathways to be significant, differences in the results are regarded due to the inherent use of different null hypothesis by each method. Additionally, as each test projects on different aspects of the data, they are complementary, increasing statistical power in the analysis of biological data, compared to individual use of any particular test (Glazko and Emmert-Streib, 2009).

It is not our goal to propose yet another classification for these methods. Instead we direct attention to the one proposed by Khatri and collaborators (Khatri et al., 2012), and keep elaborating over it. This is based on the type of analysis different PA methods perform. Main differences between classes abound in the input datasets, as well as in the analysis computations they carry out. An extensive but non-exhaustive collection of PA methods with their respective references and websites can be found in Supplementary Table 1. On what follows we shall describe a chronologically-sorted list of PA methods: over-representation analysis, functional class scoring and pathway topology-based analysis.

#### 3.2.1. First generation. over representation analysis

The basic hypothesis in an over representation analysis (ORA), is that relevant pathways can be detected if the proportion of differential expressed genes, within a given pathway, exceeds the proportion of genes that could be randomly expected.

**Figure 3:**
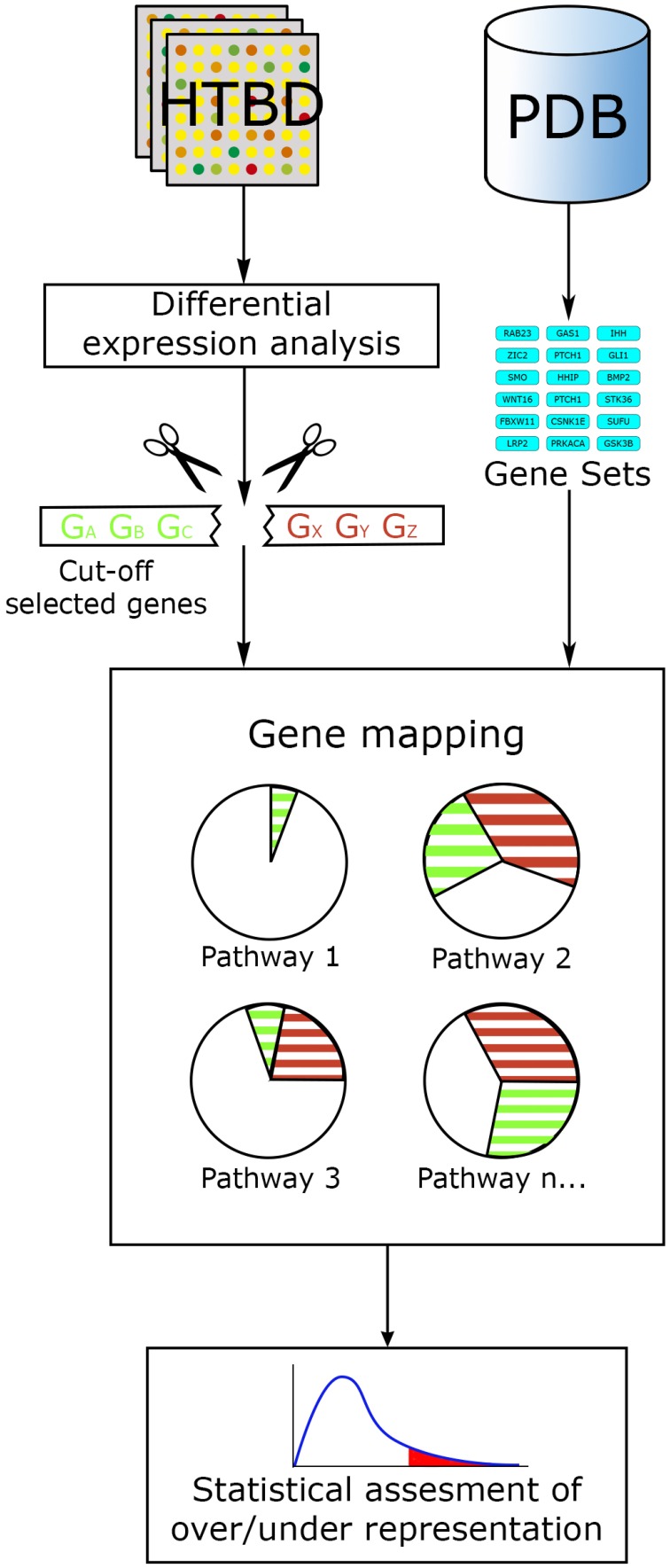
**ORA general workflow**. The main input for ORA methods is information from HTBD in the form of cut-off lists derived from expression analysis, and the pathway data in gene set format. Selected genes are mapped in the pathways, and statistical assessment of each pathway is performed using different tests.

In this way, ORA methods act along the main workflow of statistically evaluating the fraction of pathway components found among a user-selected list of biological components. This list generally fulfills certain criteria, commonly: log fold change, statistical significance or both, ranking and cutting-off the majority of components from an original list, for example, all genes tested in a microarray experiment. Then a confidence value is calculated using statistical methods such as the hypergeometric distribution, chi-square, binomial probability or the Fisher's exact test, etc., ranking pathways from the lowest *p*-value to the largest. Additional correction for multiple testing is generally performed, since evaluating data with several hypotheses simultaneously (in this case pathways) can lead to false positives. The final result from an ORA method generally consists in a list of the most relevant pathways, ordered in accordance to a *p*-value and/or a multiple-hypothesis-test-corrected *p*-value (Figure 3).

The main advantages of using ORA methodologies over non-knowledge-driven (i.e., purely data-driven) analysis is that gives Omic data a biological context, allowing to formulate hypothesis and subsequently test them experimentally, thus easily turning into a knowledge generation cycle, proper of the Systems Biology approach.

One of the most cited ORA methods is GoMiner (Zeeberg et al., 2003), which was developed for interpretation of gene-expression microarray data. It takes a list of genes that are over and under-expressed, plus the total set list of the microarray used as input, then calculates over-representation, and under-representation for Gene Ontology categories using Fisher's exact test.

Another example of ORA is WebGestalt (Wang et al., 2013), a web-based tool first published in 2005 but continually updated, (last up-date in May 2014). It performs on the basis of integrating the ORA with several central public PDBs in an interactive user-friendly platform. In this way, the method enables data analysis on different biological contexts: metabolic, Gene Ontology, gene-phenotype, gene-disease, gene-drug association, etc.

WebGestalt was used by Dopico and colleagues to inspect the functions of seasonal genes (Dopico et al., 2015). Their work found 10 gene modules which were analyzed using the biological pathways from the KEGG PDB. The analyses found that these modules are related to biological pathways of response to bacterial infections, RNA processing, metabolic pathways, and B-cell receptor signaling. Dopico et al. propose an influence of this seasonal gene expression signatures with the adaptation of human immune response. Using an ORA method like WebGestalt, enabled to give immediate meaning to the list of genes that were meticulously filtered. But in this particular example, given the time-series nature of the data, no PA method is directly capable of analyze the timed-data taking into consideration the correlations they may possess. In this case ORA, as being one of the most simple PA method class, it can be more easily tailored to analysis contexts in which gene expression or additional data would be difficult to integrate. Still the use of ORA for this kind of analysis reflects the lack of PA methods that can input time-series data directly into their workflows.

However, although ORA is efficient at rapidly identifying major biological meaning among large data sets, these methods have several limitations:

1. These methods set aside a large quantity of basal level information, due to the user selected cut-off method. Often, potentially important components close to the cut-off threshold are omitted in the analysis. This also has repercussion in results stability, as different cut-off methods yield different results (Pavlidis et al., 2004) and selection of the cut-off thresholds is arbitrary, there is no rule of thumb for establishing a cut-off threshold.
2. They evaluate every component in the pathway giving them equal weight or importance, discarding any information inherent to the interactions, (e.g., gene expression level, position in pathway, interaction between genes). In this way analysis of two pathways with the same genes but different topologies would yield the same result (Khatri et al., 2012).
3. They assume that pathways are independent to each other, which is contrary to the acknowledgment of the interaction and overlapping between pathways (Barabasi and Oltvai, 2004).

Looking for a more precise modeling of biological systems, PA has then to evolve and develop new algorithms that addressed these limitations. This led to the second generation of PA methods.

#### 3.2.2. Second generation. functional class scoring

These methods work under the main hypothesis that not only large changes in gene expression have significant effects on a pathway, but also lesser but coordinated changes in the genes that assemble the pathway have an impact on the overall pathway state. In this way Functional Class Scoring (FCS) methods use all the available measurements in HTBD to evaluate their enrichment scores, discarding the ORA cut-off threshold limitation, but still using pathways as gene sets to perform their computations.

**Figure 4:**
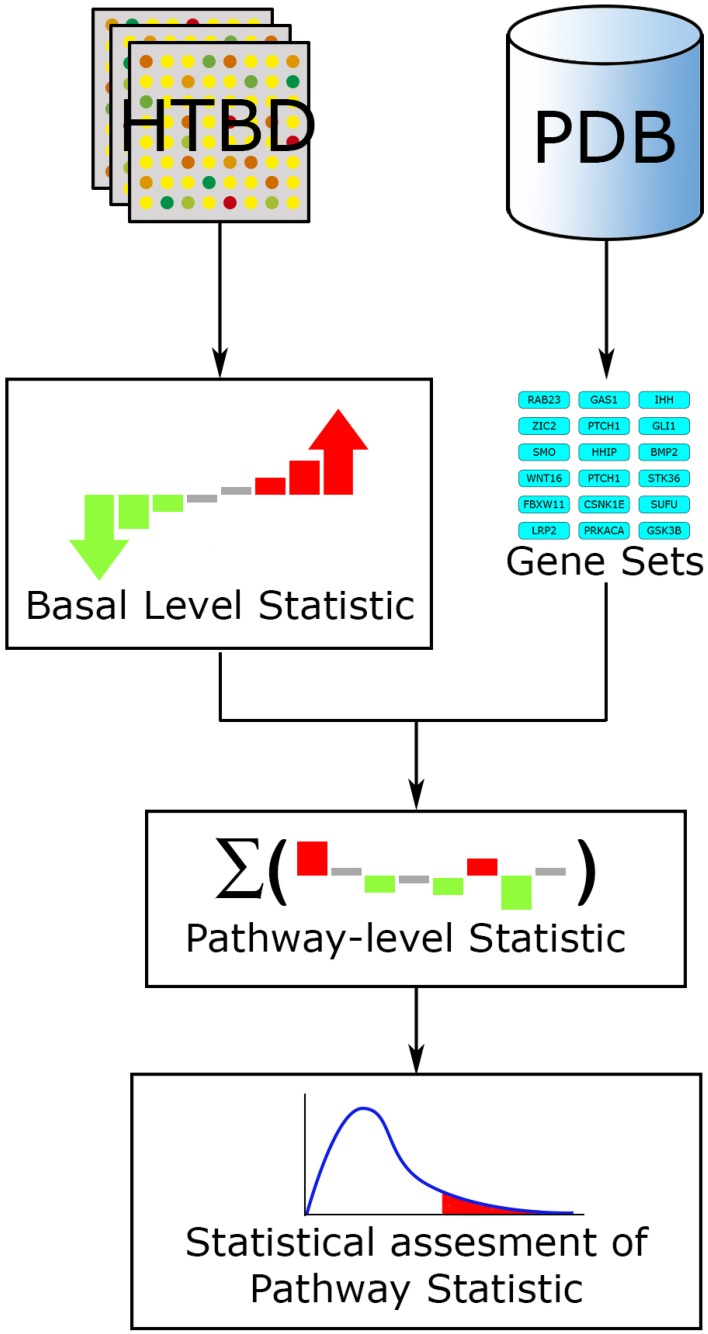
**FCS general workflow**. The main input for FCS methods are the HTBD and the pathways extracted from PDBs, in gene set format. All HTBD is used to calculate the basal level statistics, giving each component a value dependent of its differential expression. After this, the basal-level statistics of the components of each pathway is aggregated in a pathway-level statistic. Finally statistical assessment of the pathway-level statistics is performed.

Fundamentally every FCS method works along a three-step workflow (Figure 4). (1) A basal-level statistic is calculated using all the HTBD, computing differential expressions of individual components. Commonly used basal-level statistics in PA are: fold change (Qureshi and Sacan, 2013), *t*-statistic (Boorsma et al., 2005), log-likelihood ratio (Edelman et al., 2006), and signal-to-noise ratio (Subramanian et al., 2005). When sample size is small, regularized versions of these test statistics is preferred (Ackermann and Strimmer, 2009). (2) After this, the basal-level statistics from the components of each pathway, are aggregated into a single pathway-level statistic. Examples of pathway-level statistics are: Kolmogorov-Smirnov statistic (Subramanian et al., 2005), Wilcoxon sum rank statistic (Barry et al., 2005), max-mean statistic (Efron and Tibshirani, 2007), and χ^2^ (chi-squared) test (Irizarry et al., 2009). (3) Ultimately the statistical significance of the pathway-level statistics is assessed according to the selected null hypothesis. If we recall the three null hypotheses defined in Section 3.1.1, Q1 is linked to a gene sampling methodology, while Q2 is to sample label permutation, and Q3 is to re-standardization strategy (Ackermann and Strimmer, 2009).

The fundamental advantages of FCS methods, over ORA are that:

1. They use all available information and do not need an arbitrary cut-off threshold of differentially expressed genes.
2. They can detect differences between pathways that are barely passing the differentially expressed thresholds and the ones that are passing them with significance levels several orders of magnitude.
3. They can detect subtle but coordinated associations between gene-expression levels of molecules and their belonging pathways.
4. Some methods can even identify the most relevant genes in any given pathway; for instance, GSEA calls these genes the core of the pathway.

One of the first and most popular methods deploying the FCS approach is the Gene Set Enrichment Analysis (GSEA; Subramanian et al., 2005), which was developed for gene expression analysis from microarray data. In a nutshell, it uses a list of ranked genes in accordance to their differential gene expression between two phenotypic classes (by signal-to-noise ratio basal-level statistic). Then evaluates their distribution on a priori defined set of genes, (i.e., gene sets from the MSigDB) thus defining an enrichment score (ES) for each set of genes (through a Kolmogorov-Smirnov pathway-level statistic). Afterwards significance of the ES and adjustment for multiple hypothesis testing is assessed.

As an example of the methodology, the work of Folger et al. (2011) used the GSEA methodology to validate the proliferative role of growth-supporting genes they predict to be of interest in cancer treatment. In this study, the use of a gene-set external of any PDB is highlighted, as it is derived from the shRNA screening performed by Luo and colleagues in 12 cancer cell lines (Luo et al., 2008). Notably, the use of external pathway data is exploited primarily in ORA and FCS methodologies, as topology information is hard to ascertain, and is primarily obtained from PDB's crowd knowledge. Nevertheless, pathway information relative to the cell-cycle and proliferation are already available through most PDBs as KEGG and Gene Ontology.

Nonetheless, limitations to the GSEA approach were evident as soon as its first version was released (Damian and Gorfine, 2004) resulting in corrections and updates from the developers (Subramanian et al., 2005), and other groups, further extending and developing the method (Efron and Tibshirani, 2007; Jiang and Gentleman, 2007).

**Figure 5:**
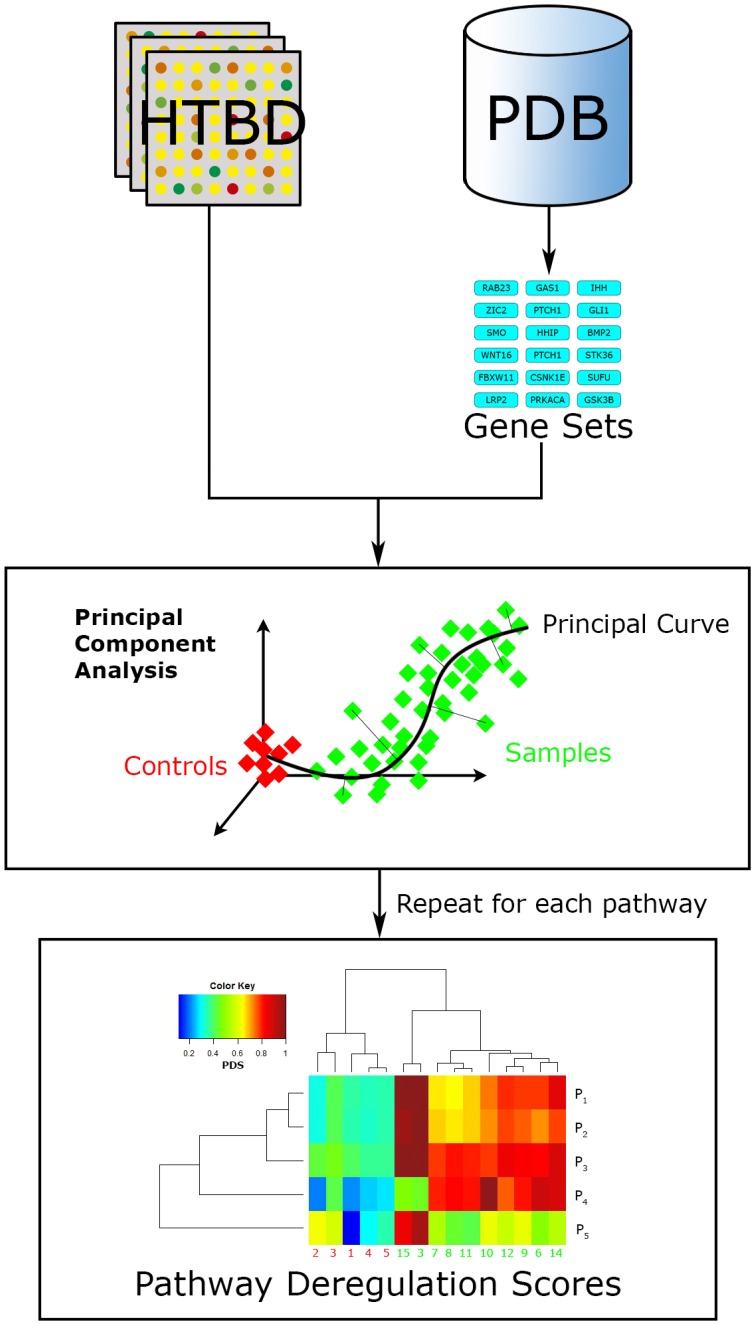
**Pathifier workflow**. Pathifier needs two inputs, a list of pathways in the gene set format, and HTBD labeled for two groups (controls vs. samples). It analyzes HTBD one pathway at a time. In this manner it gives a PDS for each sample-pathway pair, resulting in a matrix that can be examined through data driven approaches, in this case a hierarchical clustering analysis.

More recently developed, Pathifier (Drier et al., 2013) is another FCS relevant algorithm (Figure 5). It seeks to integrate the HTBD of each sample with pathway information, into a compact and biologically relevant representation, a Pathway Deregulation Score (PDS). The representation of samples in a pathway-focused manner can ultimately be useful for insight extraction. This method differs to other FCS in that it does not calculate a basal-level statistic *per se*.

Doing a brief summary, Pathifier evaluates HTBD one pathway at a time performing a principal component analysis (PCA; Pearson, 1901) over it, reducing dimensionality and noisy genes effect. Then it localizes every sample in the resulting coordinate system according to their HTBD forming a cloud of points. Afterwards a principal curve, that captures the overall sample variation, is calculated using Hastie and Stueltzle's algorithm (Hastie and Stuetzle, 1989), assigning as initial point the centroid of the control group. Finally the sample points are projected to their closest point to the principal curve, and assigned a PDS respective to their distance along the curve to the initial point (Drier et al., 2013).

Relevance of Pathifier comes from the authors acknowledgment of gaps in knowledge from PDB, therefore is constructed as a knowledge-data-driven hybrid approach. In this way it copes with the informational gaps that PDBs still have in pathway topology data. One of the greatest differences of Pathifier to other FCS is that its results are not a list of relevant pathways, but a score for each sample-pathway pair in comparison with a group of control samples.

Despite the fact that FCS analyses address limitations from ORA methods, they still carry with some issues. Mainly due to the use of pathways as gene-sets and not as networks. Examples of such limitations are:

1. Most of them still give all the components in the pathway the same weight to determine the pathway statistic—as the first release of GSEA did—, independently of *a priori* knowledge of the pathway (Khatri et al., 2012).
2. These methods still under-use the information from many PDBs as they do not take the relationships between pathways components into account, as well as other information regarding the network structure of the pathways. This can lead into diminished detection of relevant pathways (Draghici et al., 2007).
3. The methods still analyze pathways independently from each other, not accounting for overlapping between them and the influence that a pathway can exert over another (Barabasi and Oltvai, 2004).

#### 3.2.3. Third generation. pathway-topology based

Following advancements in pathway annotation from PDBs, topology of the underlying networks of pathways has been made publicly available through different databases, following immediate integration into PA methodologies. This was greatly encouraged by the increased attention network theory had in life sciences (Amaral and Ottino, 2004; Emmert-Streib and Dehmer, 2011).

The key hypothesis of pathway topology based (PTB) analysis is that interactions found in pathway topology, annotated in PDBs, bear information for interpreting correlated changes between pathway components. PTB methods can be seen as extensions of the ORA and FCS methods, as in general they perform along the same general steps, but they add pathway topology for assessing statistical relevance of the pathways.

**Figure 6:**
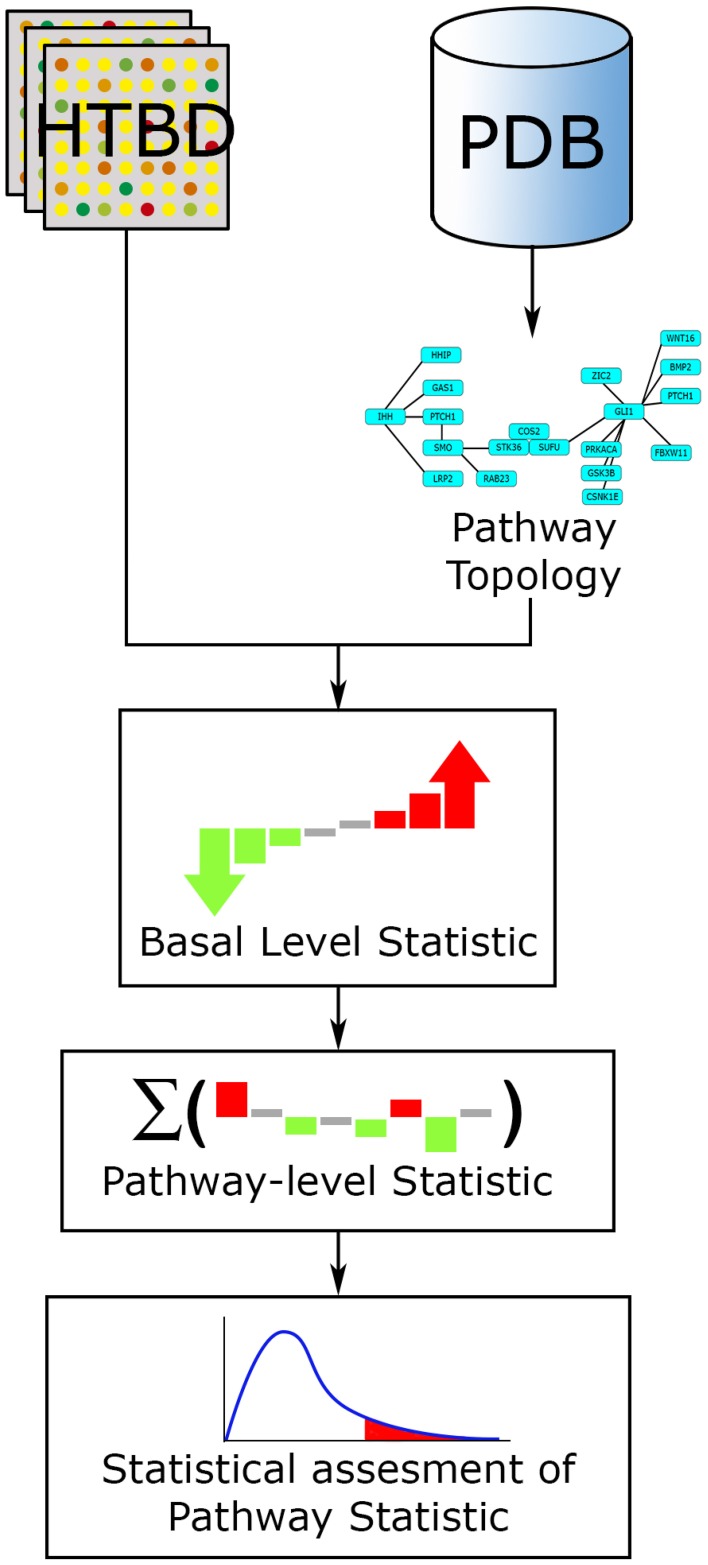
**PTB analysis general workflow**. The main inputs for PTB methods are the HTBD and the pathways extracted from PDBs, in pathway topology format. All HTBD and pathway topology is used to calculate the basal-level statistics. After this, the basal-level statistics of the components of each pathway is aggregated into a pathway-level statistic. Finally statistical assessment of the pathway-level statistics is performed.

In the case of ORA extended methods, user selected genes are mapped on the pathway topology and subsequent network and statistical analyses are performed. In the case of the FCS extended methods, the HTBD along with the topology is used to compute the basal-level statistics, and proceed further in a similar way to a FCS approach (Khatri et al., 2012; Figure 6).

By analyzing pathways as networks, PTB analysis cope with limitations from the ORA and FCS methods as:

1. Based on topology information they can account for biologically relevant differences between components, by giving more weight to changes in genes with greater influence over the pathway.
2. By taking into account different topological information, they enable a more precise analysis of a same set of pathway components, as some interactions may be known to be different under certain biological conditions.
3. Account for causal interactions within the pathways, as modifications in upstream components are expected to change the behavior of downstream components.

One of the earliest methods that implemented pathway topology in its analysis was Pathway-Express, as part of the Onto-tools suite (Khatri et al., 2005; Draghici et al., 2007). Inspired by the sentiment that as more data become available, the question “Is there a known pathway containing my gene(s) of interest?” would transform into “How do I find the most interesting pathway(s) involving my gene(s)?”

This method calculates two probabilistic terms, (1) the gene perturbation factor, which uses a similar approach to the Page-Rank index used by Google (Page et al., 1999), taking into account the normalized fold change of each input gene, and the amount of perturbation of genes downstream it, reflecting the relevance of each differentially regulated gene; and (2) the impact factor of the entire pathway, which takes into account the gene perturbation factors of all the genes in each pathway, and the proportion of differentially regulated genes on it. Finally presenting a ranked list of pathways according to their impact factor and multiple-hypothesis testing correction as given by its false discovery rate statistic.

More recently, the same developing team of Pathway-express developed the Signaling Pathway Impact Analysis (SPIA; Tarca et al., 2009), which is an improved version of the first. The impact analysis of SPIA seeks to determine and combine two types of evidence, assuring independence between the probabilities of each one: (1) the over representation of differentially expressed genes in a given pathway, and (2) the abnormal perturbation of the pathway, measured by propagating expression changes across the pathway topology.

Another established PTB methodology is PARADIGM (Vaske et al., 2010), which has been developed to integrate any number of Omic datasets to infer pathways altered in a patient-specific- or sample-specific-manner. It uses a probabilistic graphical model framework for learning the underlying causal networks compliant with the provided observations from HTBD.

In their model-testing, Vaske and collaborators used copy number variation and gene expression data from a glioblastoma dataset, along with the pathway topology information from the National Cancer Institute Pathway Interaction Database (NCI-PID), to successfully stratify the patient samples into clinically relevant clusters. PARADIGM, as well as the previously mentioned methodology Pathifier (Drier et al., 2013), do not provide a list of relevant pathways, but give a score about each sample status paired to every analyzed pathway. This score in the PARADIGM methodology is called the Integrated Pathway Activity (IPA) score—not to be confused with Ingenuity Pathway Analysis—. Thus, the final result of such an analysis is a matrix, where columns correspond to samples and rows to pathways, and each value in it, is calculated for each Sample-Pathway pair. With this matrix, a clustering analysis can be performed, grouping samples in molecular subtypes by pathway-relevant means, instead of a more traditional gene-wise approach.

An illustrative use of the PTB methods is the work from Heiser and colleagues, in which they used PARADIGM to determine if cell lines were similar to tumor samples of the same subtype (Heiser et al., 2012). In this study, the pathway information from the PDBs BioCarta, Reactome, and NCI-PID, as well as gene expression and copy number data, were used as input. A potential caveat for this study is inherent to PTB methods limitations, is the gap that crowd knowledge may have on the topology of pathways which limits the pathways and PDBs to be analyzed, to the ones with available topology information.

Limitations to PTB methodologies are hard to address as they portray a change in one of the current paradigms in life sciences. Life components do not work isolated, functioning by their own, they rather do so in precise concert to accomplish life functions, and their behavior as a whole system is dynamic, adaptable and robust (Hartwell et al., 1999; Tononi et al., 1999; Amaral and Ottino, 2004). In summary, life is a complex system. However, some limitations of PTB methods can be pointed out, that will certainly be addressed in future methods, as experimental and annotation barriers are surmounted:

1. Some PTB methods do not recognize direction of the connections between the pathway components. Chain effects of deregulation can be missed (Tarca et al., 2009).
2. PTB methods do not take into account the interconnections between pathways, for improving detection of relevant pathways. Take for instance that, downstream components in “pathway A” can be upstream components in “pathway B,” therefore it can be expected that pathway A has influence over pathway B (Yaffe, 2008).
3. There is a lack of consideration about time and spatial distribution for pathway components in their models. Pathway behavior may be dependent of biomolecule compartmentalization, for instance: transcriptional regulation in the nucleus, protein transport in endoplasmic reticulum and mitochondria mediated signaling. Pathway standard languages such as BioPAX, SBGN, and SBML already support compartments in their pathway ontologies (Hucka et al., 2003; Le Novere et al., 2009; Demir et al., 2010).
4. Additionally, molecular regulation in a time-scale manner is also relevant to understand the mechanisms by which pathways are working in the cell. As technology becomes cheaper, experimental costs should drop and time scale analyses would become more frequent, thus increasing the need for better PA tools able to analyze such data (Bar-Joseph et al., 2003).
5. Most methods cannot recognize the multiple states and variants that a pathway component can have. For instance, most PA methods collapse splicing variants from gene expression data to a single HGNC gene symbol. Improvement in this regard should acknowledge for biologically relevant information as: single nucleotide polymorphism (SNP) variants, splicing variants, epigenetic modifications and postranslational modifications, as well as their potential influence in phenotype and pathway functioning (Khatri et al., 2012).

## 4. Challenges and perspectives

There are still many challenges in the development and usage of PA methods as well as in the refinement of their foundations. Similarly to the previous division of introductory topics, the challenges can be approached in three categories: improvements in Omics technologies, annotations of PDBs, and of PA methods development and usage, according to the progression of their foundations.

### 4.1. Omic technologies

As costs minimize and coverage of different sets of biomolecules increases, PA methods should develop toward more integrative models of biology, such as the one employed in the PARADIGM methodology (Vaske et al., 2010) in which different kinds of Omic data can be integrated to increase the detection of pathways in each sample. In line with Bayes' theorem, in which additional evidence from an event updates the probability ratios, in such a way that posteriors improve substantially over priors, the addition of complementing Omic datasets should enrich the information of any study, and therefore improve its analysis through PA.

Time scaled data and subcellular fractionation experiments are also expected to give more precise insight into how are pathway components interacting. Information derived from such experiments should be included into both the annotations of PDBs, as well as in the analytical capabilities of PA methods.

Since PA methods have developed for over little more than a decade, its common analysis targets are microarray data and SNP data, still overlooking new technologies. Next generation sequencing technologies (NGS) are becoming a new standard in modern life sciences research, demand of analytical techniques developed for NGS data analysis is growing. RNA sequencing (RNA-seq), is a NGS technology that generates data not only of the expression levels of the RNA transcripts in a tissue (or cell), but also data regarding sequence polymorphisms and structure of the RNA templates. Using standard PA methods to analyze RNA-seq data may give biased results due to over detection of differential expression for long and highly expressed transcripts (Oshlack and Wakefield, 2009). An FCS method that seeks to address this issue is GOseq (Young et al., 2010), using Gene Ontology as its main PDB. Another FCS method that addresses the previous biases is SeqGSEA (Wang and Cairns, 2014), and additionally integrates splicing data into its analysis, addressing the sequence polymorphisms, afterwards it implements the traditional methodology of GSEA. To our knowledge there is no PA methods that directly inputs epigenome or chromatin immunoprecipitation sequencing (ChIP-seq) data into their workflows. As NGS technologies continue to grow in usage, development of tools that can integrate their wealth of data will be of great advantage toward reproducibility of research, as current pathway-oriented analysis of these datasets may use tailor-made methodologies.

### 4.2. PDBs annotation

As mentioned before, there is no agreement as to a universal definition of pathways, nor a unified definition and usage of a pathway ontology. Perhaps this was primed by the biological focus each separate project had in its beginnings, or as a result of cellular functions being too disparate to be contained within a single paradigm. However, unification of similar ontologies and usage of standard languages should prove to be the most pragmatic and viable solution, across PDBs and PA methodologies.

Annotation coverage should also be increased. As mentioned before, more than half of the symbols used by the HGNC project have no allocation within any pathway on the PDB Pathway Commons (Cerami et al., 2011), one of the biggest composite databases. This fact leaves a great potential working space for manual curation, computer-driven annotation and experimental discovery. Additionally, procedures such as, including as publishing requirements the annotation of experimental results into PDBs employing pathway standard languages, may drive annotation efforts forward.

Assert interconnection between pathways is a reasonable yet difficult task, as different ontologies of pathways may lead to different result to ascertain the connection between pathways (Green and Karp, 2006), yet it is evident that for an integrative model of biology, this connections should be taken into consideration (de Anda-Jáuregui et al., 2015).

### 4.3. PA methods development and usage

As diversification of PA progress, selection of suited methods becomes daunting, since evaluation standards for these methods do not exist yet. One is generally given the option to employ the most popular and well-understood methods, which in some cases may not be optimum.

Difficulties in the generation of such evaluation standards may come from the fact that the “absolute truth” about the state of pathways in real experiments is not ultimately known. Thus, asserting statistical performance for precision, recall and specificity is not obvious. Additionally, some methodologies are certainly different in statistical and mathematical approaches employed, making them not comparable between them.

Shedding some light into this problem, Tarca and collaborators pointed out that in the absence of gold standards for evaluation, the best alternative is to compare between methods in the light of the existing biological knowledge (Tarca et al., 2009). Also Mitrea and collaborators suggest the design of benchmark data for asserting the statistical power of the methods (Mitrea et al., 2013), but this has proven to be a difficult task.

Regarding the output formats, it is important to call attention to the ones provided by PA methods such as ASSESS (Edelman et al., 2006), Paradigm (Vaske et al., 2010), and Pathifier (Drier et al., 2013). Such outputs are composed fundamentally of a matrix of paired scores, which integrate the PA for each sample within the space of the analyzed samples. This in turn, can give a representation of how different is the functioning of every pathway in every sample, in contrast with the control group.

Representation of molecular functioning status in a sample-pathway perspective may prove to be helpful, and can provide insight into functional molecular heterogeneity, as between samples grouped under the same phenotype, but with different molecular origin. For instance, cancer, in which there are different malfunctioning pathways behind the disease main characteristics, converging in similar phenotypes (Hanahan and Weinberg, 2011).

Since these scores depend on single sample measurements, embedded in the sample space, statistical certainty is difficult to estimate. Discussion on methods to verify the confidence of these scores is thus needed. Nevertheless, advancements in discussion and improvements of such methodologies, may help to develop a new generation of PA methods, in which every sample is on the focus, instead of the overall sample-set, extending the now existing methods toward the understanding of pathway phenomena at a deeper biological level.

## 5. Concluding remarks

PA is a blooming interdisciplinary research area that is steadily growing and developing. Most current methodologies are developed to use pathway topology information stored in different PDBs and all the measurements coming from high throughput technologies, yet there are challenges that need to be surpassed for better identification of relevant pathways. Particularly relevant are the annotation depth and coverage challenge, which PDBs as collective knowledge suffer, and the need for all scientific community to collaborate in their advancement.

Attention needs to be given to newer methodologies that attempt to zoom the analysis scope to the sample scale, as they can be useful in the generation of personalized pharmacological therapy. Transformation from thousands of gene-level measurements to tens of biologically meaningful pathways in the sample scale is a desirable approach. This may facilitate further analysis and hypothesis generation at a personal level.

As already noted, PA is a very diverse set of methodologies that may help formulate more educated hypothesis about the data Omic technologies generate in any research endeavor that employs this kind of biological data retrieval approach. After all the issues we have discussed, one thing may already become evident: PA methods should never be taken as black boxes from where experimental *data goes-in*, and true *statements come-out*, but perhaps more as cleaners of haystacks from where we are pursuing to find biological meaningful needles.

## Author contributions

MAGC: Contributed to the writing of the manuscript, designed and made the figures, revised the manuscript. JEE: Contributed to the writing of the manuscript, designed the figures, revised the manuscript. EHL: Conceived the idea, contributed to the writing of the manuscript, revised the manuscript.

## Funding

CONACyT (grant no. 179431/2012), (grant no. 232647/2014).

### Conflict of interest statement

The authors declare that the research was conducted in the absence of any commercial or financial relationships that could be construed as a potential conflict of interest.
