# Supplementary material for: Pathway Analysis: State of the Art
Source: Front Physiol. 2015 Dec 17;6:383. doi: 10.3389/fphys.2015.00383 (PMC4681784; doi:10.3389/fphys.2015.00383)
Supplement: Supplementary file 1 [file Table1.pdf]

ORA				
**Method**	**Implementation URL**	**Reference**	**Google Scholar cites until August 2015**	**Year of release**
FunSpec	funspec.med.utoronto.ca	Robinson et al., 2002	342	2002
Onto-Express	vortex.cs.wayne.edu/ontoexpress	Khatri et al., 2002	427	2002
EASE	david.abcc.ncifcrf.gov/ease/ease1.htm	Hosack et al., 2003	1747	2003
FuncAssociate	llama.mshri.on.ca/funcassociate	Berriz et al., 2003	377	2003
GARBAN	garban.tecnun.es/ (NLA)	Martínez-Cruz et al., 2003	29	2003
GeneMerge	cbcb.umd.edu/software/genemerge (NLA)	Castillo-Davis et al.,2003	258	2003
GoMiner	discover.nci.nih.gov/gominer	Zeeber et al., 2003	1147	2003
MAPPFinder	genmapp.org/help_v2/MAPPFinder.htm	Doniger et al., 2003	922	2003
FatiGO	babelomics.org	Al-Shahrour et al., 2004	1002	2004
GO:TermFinder	search.cpan.org/dist/GO-TermFinder	Boyle et al., 2004	978	2004
GOstat	gostat.wehi.edu.au	Beißbarth et al., 2004	1021	2004
GOToolBox	genome.crg.es/GOToolBox/	Martin et al., 2004	329	2004
Ontologizer	compbio.charite.de/ontologizer	Robinson et al., 2004	100	2004
WebGestalt	bioinfo.vanderbilt.edu/webgestalt	Zhang et al., 2005	790	2005
BiNGO	psb.ugent.be/cbd/papers/BiNGO/Home.html	Maere et al., 2005	1548	2005
GOFFA	edkb.fda.gov/webstart/arraytrack	Sun et al., 2006	52	2006
GOStats	bioconductor.org	Falcon & Gentleman, 2007	734	2007
GOEAST	omicslab.genetics.ac.cn/GOEAST	Zheng et al., 2008	379	2008
ClueGO	apps.cytoscape.org/apps/cluego	Bindea et al., 2009	459	2009
agriGO	bioinfo.cau.edu.cn/agriGO	Du et al., 2010	594	2010
GO-Bayes	Upon author request	Zhang et al., 2010	20	2010
GO-Elite	genmapp.org/go_elite/	Zambon et al., 2012	58	2012
**FCS**				
**Method**	**Implementation URL**	**Reference**	**Google Scholar cites until August 2015**	**Year of release**
Catmap	bioinfo.thep.lu.se/catmap.html	Breslin et al. 2004	68	2004
GlobalTest	bioconductor.org	Goeman et al., 2004	695	2004
GOAL	microarrays.unife.it (NLA)	Volinia et al. 2004	50	2004
GO-Mapper	gatcplatform.nl/gomapper/	Smid et al. 2004	57	2004
IGA	biomedcentral.com/1471-2105/5/34	Breitling et al., 2004	143	2004
Erminej	bioinformatics.ubc.ca/ermineJ	Lee et al., 2005	234	2005
GSEA	broadinstitute.org/gsea	Subramanian et al., 2005	7840	2005

FunCluster	corneliu.henegar.info/FunCluster.htm	Henegar et al., 2006	20	2005
PLAGE	dulci.biostat.duke.edu/pathways/ (NLA)	Tomfohr et al., 2005	147	2005
SAFE	bioconductor.org	Barry et al., 2005	253	2005
sigPathway	bioconductor.org	Tian et al., 2005	497	2005
T-profiler	t-profiler.org	Boorsma et al., 2005	191	2005
AE	No implementation available	Saxena et al. 2006	40	2006
ASSESS	people.genome.duke.edu/assess/	Edelman et al. 2006	64	2006
JProGO	jprogo.de/	Scheer et al. 2006	38	2006
Category	bioconductor.org/packages/Category/	Gentleman et al., 2007	165	2007
Fatiscan	babelomics.org	Al-Shahrour et al. 2007	112	2007
GAzer	integromics.kobic.re.kr/Gazer (NLA)	Kim et al., 2007	33	2007
GeneTrail	genetrail.bioinf.uni-sb.de	Backes et al., 2007	239	2007
GlobalANCOVA	bioconductor.org	Hummel et al., 2007	108	2007
GSA	cran.r-project.org/web/packages/GSA	Efron et al. 2007	539	2007
PCOT2	bioconductor.org/packages/pcot2/	Song et al., 2007	73	2007
Allez	stat.wisc.edu/~newton/	Newton et al. 2007	167	2007
SAM-GS	ualberta.ca/~yyasui/homepage.html	Dinu et al., 2007	207	2007
FUNC	func.eva.mpg.de/	Prüfer et al., 2007	91	2007
Eu Gene Analyzer	duccioknights.org/?page_id=169	Cavalieri et al. 2008	39	2008
GAGE	bioconductor.org	Luo et al., 2009	130	2009
MGSA	bioconductor.org	Bauer et al., 2010	98	2010
MSEA	msea.ca/MSEA/faces/Home.jsp	Xia et al., 2010	96	2010
GOSeq	bioconductor.org/packages/goseq/	Young et al., 2010	335	2010
MD-GSA	bioconductor.org/packages/mdgsa/	Montaner & Dopazo, 2010	20	2010
ADGO 2.0	btool.org/ADGO2	Nam et al. 2011	6	2011
Pathifier	bioconductor.org	Drier et al., 2013	39	2013
GSVA	bioconductor.org/packages/GSVA/	Hänzelmann et al., 2013	40	2013
SeqGSEA	bioconductor.org/packages/SeqGSEA/	Wang and Cairns, 2014	7	2014
GSAASeqSp	gsaa.unc.edu/	Xiong et al., 2014	5	2014
PathwaySeq	Code provided in publication page	Zhou, 2015	0	2015
**PTB**				
**Method**	**Implementation URL**	**Reference**	**Google Scholar cites until August 2015**	**Year of release**
ScorePAGE	No implementation available	Rahnenführer et al., 2004	93	2004
IPA *	ingenuity.com	Calvano et al., 2005	1080	2005
Pathway-Express	vortex.cs.wayne.edu/projects.htm	Khatri et al., 2005	111	2005

MetaCore *	genego.com	Nikolsky et al., 2005	112	2005
WPS	abcc.ncifcrf.gov/wps (NLA)	Yi et al., 2006	133	2006
MATISSE	acgt.cs.tau.ac.il/matisse	Ulitsky and Shamir, 2007	244	2007
TAPPA	watson.mcgee.mcw.edu:8080/~sgao (NLA)	Gao and Wang, 2007	19	2007
Ontologizer 2.0	http://compbio.charite.de/contao/index.php/ontologizer2.html	Bauer et al., 2008	277	2008
NetGSA	cran.r-project.org/package=netgsa	Shojaie and Michailidis, 2009	33	2009
PIPA	No implementation available	Bankhead et al., 2009	13	2009
SPIA	bioconductor.org	Tarca et al., 2009	241	2009
Snow	babelomics.org	Minguez et al., 2009	34	2009
DEGraph	bioconductor.org/packages/DEGraph/	Jacob et al., 2010	18	2010
MetPA	metpa.metabolomics.ca	Xia and Wishart, 2010	86	2010
PARADIGM	sbenz.github.io/Paradigm	Vaske et al., 2010	235	2010
PWEA	zlab.bu.edu/PWEA	Hung et al., 2010	34	2010
TopoGSA	topogsa.org	Glaab et al., 2010	26	2010
TopologyGSA	cran.r-project.org/package=topologyGSA	Massa et al., 2010	32	2010
BPA	bioinfo.unl.edu/bpa	Isci et al., 2011	18	2011
DART	bioconductor.org	Jiao et al., 2011	11	2011
GANPA	cran.r-project.org/package=GANPA	Fang et al., 2011	12	2011
Pathologist	ftp://ftp1.nci.nih.gov/pub/pathologist	Greenblum et al., 2011	15	2011
ACST	dx.doi.org/10.1371/journal.pone.0041541	Mieczkowski et al., 2012	7	2012
CePa	cran.r-project.org/package=CePa	Gu et al., 2012	5	2012
EnrichNet	http://www.enrichnet.org	Glaab et al., 2012	56	2012
PathNet	bioconductor.org	Dutta et al., 2012	14	2012
THINK-Back-DS	wwweb.eecs.umich.edu/db/think/	Farfán et al., 2012	6	2012
Network Miner	babelomics.org	García-Alonso et al., 2012	16	2012

Supplementary Table 1: List of found Pathway Analysis methods available. The first column gives the names of the methods. The second one is the web site to each method. The third column contains the original reference to each method. The fourth column contains the number of citations to date of each one of those pathway analysis methods. Finally, the fifth column shows the year of release of those packages. * = Commercial PA method.
